# A Preshaped Titanium Mesh for Guided Bone Regeneration with an Equine-Derived Bone Graft in a Posterior Mandibular Bone Defect: A Case Report

**DOI:** 10.3390/dj7030077

**Published:** 2019-08-01

**Authors:** Danilo Alessio Di Stefano, Gianbattista Greco, Enrico Gherlone

**Affiliations:** 1 Dental School, Vita e Salute University and IRCCS San Raffaele, 20132 Milan, Italy; 2 Private Practice, 20148 Milan, Italy; 3 Private Practice, 20090 Trezzano sul Naviglio, Italy

**Keywords:** case report, guided bone regeneration, titanium mesh, xenografts, bone collagen

## Abstract

One of the most often used bone augmentation techniques is the guided bone regeneration procedure. The authors report the case of a 75-year-old man with an atrophic right posterior mandible who underwent bone augmentation through guided bone regeneration with a preshaped titanium mesh adapted on a stereolithographic model of the patient’s jaw. The graft volume was simulated with a light-curing resin. The actual site was grafted with a mixture of autogenous and equine-derived bone. Five months later, the mesh was retrieved, three cylindrical implants were positioned, and a bone biopsy was collected for histomorphometric analysis. A provisional prosthesis was delivered three and a half months later. Definitive rehabilitation was accomplished after one additional month. The graft allowed for effective bone formation (newly formed bone, residual biomaterial, and medullar spaces were, respectively, 39%, 10%, and 51% of the core volume). The patient has functioned successfully throughout six and a half years of follow-up. Using the preshaped titanium mesh in association with the enzyme-treated equine bone substitute provided effective bone regeneration.

## 1. Introduction

Prosthetically driven implantology calls for designing the optimal functional and aesthetic rehabilitation in advance by means of dedicated software programs that reproduce the patient’s anatomy, starting from computed tomography (CT) or cone-beam computed tomography (CBCT) scans. As patients requiring rehabilitation may suffer from bone atrophy, preplanned implant positions do not necessarily correspond to regions with an adequate bone quantity for implant insertion and stabilization. If severe atrophy is present, restoring the alveolar bone volume is a necessary preliminary step to perform dental implant rehabilitation in a safe and predictable manner.

One of the most widely used bone augmentation techniques is the guided bone regeneration (GBR) procedure. It involves interposing a barrier between the regenerating bone and the surrounding soft tissues, thus preventing non-osteogenic cells from entering and populating the regenerating site [1,2]. Various bone grafts have been used over the years in an attempt to enhance and facilitate bone regeneration; these include anorganic bovine bone mineral (ABBM) [3]; freeze-dried bone allograft (FDBA) [4], or FDBA in combination with autologous bone chips (ABC) [5], ABBM, and ABC with different concentrations [6]; *β*-tricalcium phosphate/hydroxyapatite (*β*-TCP/HA) [7]; demineralized bone matrix (DBM) [8]; and hydroxyapatite (HA) alone [9]. These grafts have been used in association with a variety of resorbable or nonresorbable membranes [10,11,12,13]. Nonresorbable barriers include titanium meshes [14,15,16,17,18], which provide a thorough tenting effect and optimally protect the underlying regenerating bone thanks to their rigidity. Additionally, they are easily moldable and adaptable to the shape of the defect and, once modelled, they maintain their shape over time. The shaping step is usually performed during surgery, on the defect itself, after filling it with the graft. This lengthens the surgery time, exposing the patient to additional discomfort and morbidity risk. However, meshes can be shaped in advance on stereolithographic models of the patients’ jaws, instead of during surgery, with clear advantages in terms of reduction of surgical time and simplification of the procedure.

This report describes the case of a patient who presented with an atrophic posterior mandible, was successfully treated by GBR augmentation with a preshaped titanium mesh and an equine-derived bone graft, and was followed for 6.5 years after rehabilitation.

## 2. Case Description 

**Figure 1:**
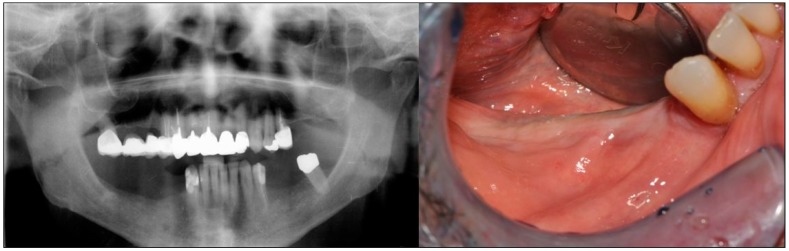
Pre-operative panoramic radiography and intraoral examination. The patient was missing from teeth 4.4 to 4.8.

**Figure 2:**
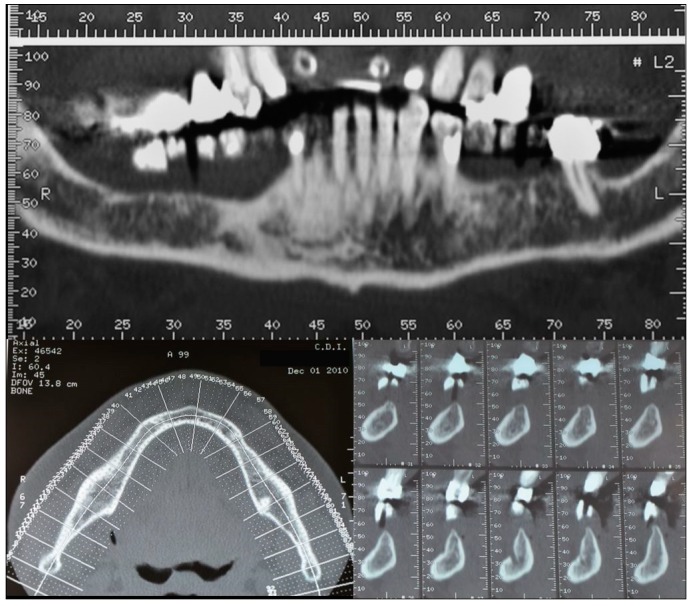
CT scans showing the horizontal atrophy in the lower right quadrant.

The patient was a 75-year-old man with a noncontributory medical history seeking to improve his masticatory function on the lower right side of his mouth. A comprehensive intraoral examination was performed to assess his overall oral health. The patient had a good oral status, with full mouth plaque score (FMPS) and full mouth bleeding score (FMBS) equal to 0 and 1, respectively. The patient was missing from teeth 4.4 to 4.8 (Figure 1); clinical and radiographic examination revealed a horizontal defect of the corresponding alveolar ridge (Figure 2) that was classified as Class IV according to Cadwood and Howell [19]. A two-step procedure that included GBR with a titanium mesh and consequent placement of three dental implants was developed.

**Figure 3:**
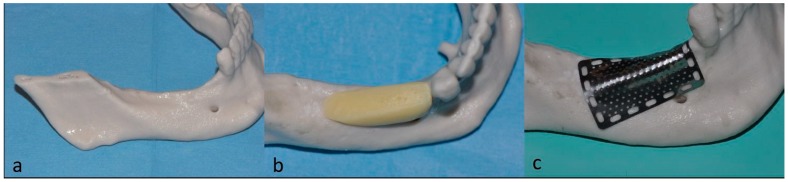
A few days before the surgery (**a**) a stereolithographic model of the patient’s lower ridge was fabricated; (**b**) a light-curing resin was applied to simulate the restored alveolar bone volume, and (**c**) the titanium mesh was adapted to perfectly conform to the jaw model.

On the basis of CBCT scans, a model of the lower ridge was prepared (Figure 3a) and used as a template to mold a light-curing resin (Fuji Ortho LC; GC America Inc, IL, USA) (Figure 3b) to simulate the restoration of the crestal bone volume. A titanium mesh (Titanguide, Prodent Italia, Pero, Italy) was then preshaped on the model (Figure 3c) and sterilized in an autoclave some days before the surgery.

Antibiotic prophylaxis (amoxicillin/clavulanic acid, Augmentin, Glaxo-SmithKline, Verona, Italy; 2 g 1 h before surgery and then every 12 h for 8 days) was initiated, and the patient was given mouth rinses with chlorhexidine 0.2% (Corsodyl, Glaxo-SmithKline) to be continued for 2 weeks after surgery. Nimesulide 100 mg (Aulin, Roche, Milano, Italy) also was administered 1 h before surgery and then twice a day for 7 days. The surgical area was anesthetized with articaine hydrochloride 1% with epinephrine 1:100,000 (Molteni Dental, Milano, Italy).

**Figure 4:**
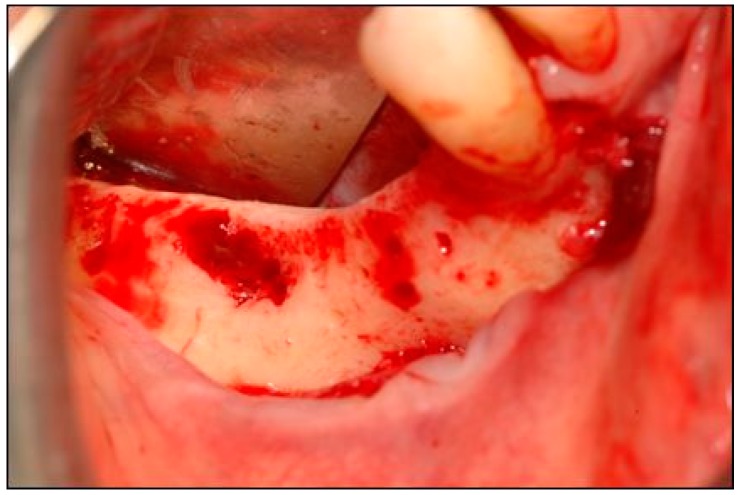
The mucoperiostial flap was elevated to expose the alveolar ridge.

A mid-crestal full-thickness incision was created and was extended to tooth 4.3 through an intrasulcular incision. Two vertical incisions were then performed to prepare a trapezoidal mucoperiosteal flap. The flap was elevated on both the vestibular and lingual sides to expose the right mandibular quadrant (Figure 4).

**Figure 5:**
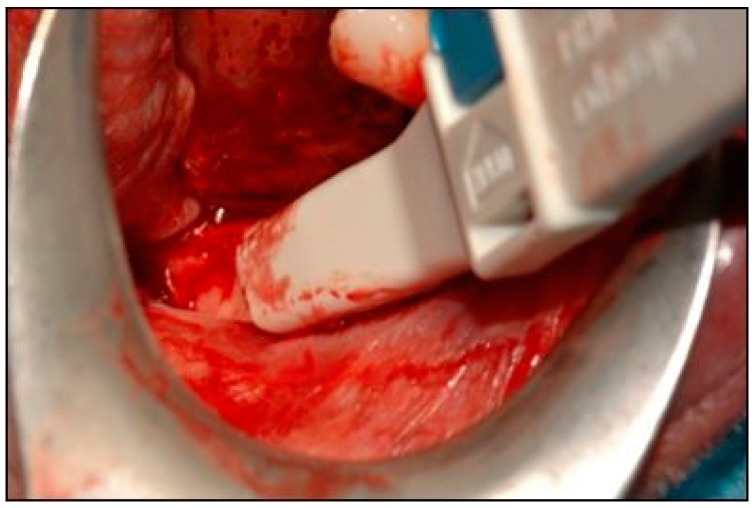
A bone scraper was used to collect autologous bone tissue.

The site was then prepared for bone augmentation with a bone scraper (Safescraper^®^ Twist, Meta, Reggio Emilia, Italy) (Figure 5) to facilitate graft vascularization and cell colonization.

**Figure 6:**
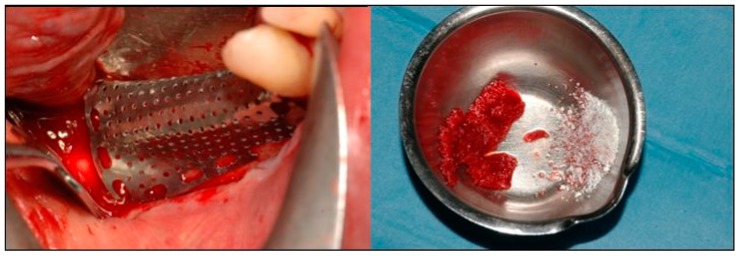
(**Left**) The fit of the preshaped mesh was checked, and (**Right**) the autogenous bone was mixed with the xenograft.

After checking the optimal fit of the preshaped mesh on the defect (Figure 6a), a mix of autogenous bone and equine enzyme-processed 0.5–1.0 mm bone granules (Osteoxenon^®^ Mix Bone Granules, Bioteck, Arcugnano, Italy) was prepared in a 50:50 ratio (Figure 6b). Autogenous bone was mixed with the xenograft to possibly exploit the bone regenerative potential of the live cells and growth factors it contains [20,21].

**Figure 7:**
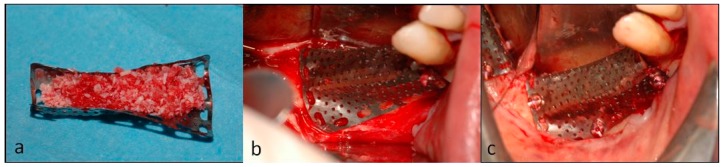
(**a**) The mesh was filled with the graft material and then (**b**) used to plaster the graft on the defect. (**c**) The mesh was fixed with mini-screws.

The mixture was placed on the bone defect using the mesh as an aid (Figure 7a). The mesh was positioned over the restored defect (Figure 7b) and fixed with mini-screws (Figure 7c).

**Figure 8:**
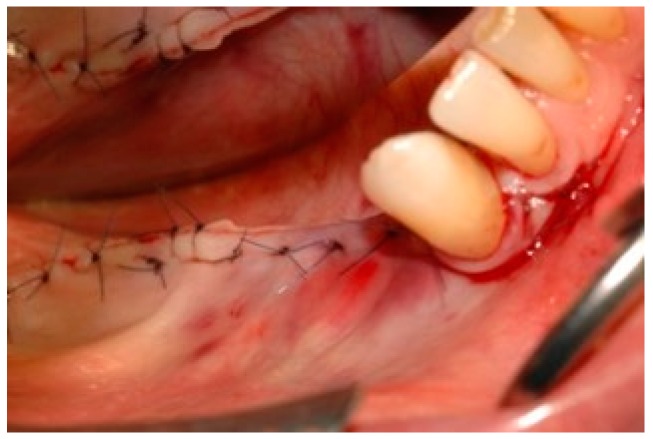
After checking the absence of any residual tension, the flap was sutured with nonresorbable suturing stitches.

After checking proper flap release, complete flap closure was achieved using a nonresorbable suture (Monomyd 4-0/5-0 Polyamide Monofilament Suture, Butterfly, Cavenago, Italy) (Figure 8). Sutures were removed after eight days.

**Figure 9:**
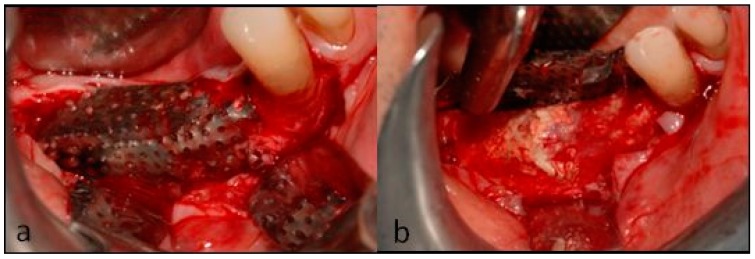
At the second surgery, (**a**) the flap was opened, and (**b**) the mesh was retrieved.

The second surgery followed five months after the GBR procedure, under the same pre- and post-surgical antibiotic prophylaxis and antalgic therapy as previously described. A flap was opened extending from position 4.3 to 4.7, the screws were removed, and the mesh was retrieved (Figure 9).

**Figure 10:**
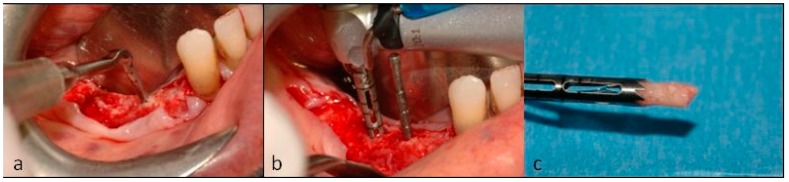
(**a**) Piezosurgery was used to prepare position 4.4 for implant placement. (**b**) The other two tunnels were drilled, and bone biopsies (**c**) were collected with a trephine.

As tissue in position 4.4 was found to be soft, the implant site was prepared using a piezosurgery insert (Esacrom S.r.l., Imola (BO), Italy, Figure 10a). The other two implant sites were drilled (Figure 10b) and bone biopsies were collected for histological evaluation. The first narrow drilling was performed with a 2.7 mm external diameter bone-collecting trephine instead of a narrow drill (Figure 10c); then the hole was drilled to its final diameter using the drilling sequence advised by the implant manufacturer. 

Given this sequence of operations, the patient was subject to no additional tissue damage and no additional treatment with respect to the standard clinical practice. Then, three cylindrical implants (Xive, Dentsply, York, PA, USA) were placed: 3.4 × 11 mm, 3.4 × 9.5 mm, and 3.8 × 11 mm in positions 4.4, 4.5, and 4.6, respectively. The insertion torque (IT) of the implant in position 4.4 was 16 Ncm; the IT values of the implants in positions 4.5 and 4.6 were 23 and 25 Ncm, respectively.

**Figure 11:**
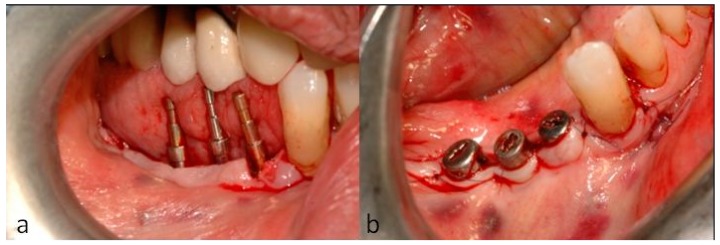
(**a**) The parallelism of the implants was confirmed, and (**b**) the soft tissue was sutured around the healing abutments.

Their alignment was verified using paralleling pins (Figure 11a) and the flaps were closed with nonresorbable sutures (Figure 11b) that were removed six days later.

**Figure 12:**
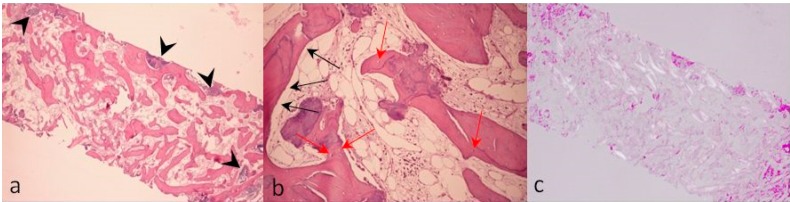
Bone biopsies collected five months after the GBR procedure were stained with haematoxylin and eosin (H&E) and examined by light microscopy (**a**, 3.5×; **b**, 10×) and under polarized light (**c**, 3.5×). Black arrows in subfigure **a** indicate the residual graft material. Black and red arrows in subfigure **b** respectively show osteoblasts and osteocytes within the newly formed bone. No osteoclasts were detected in the slides being analyzed.

Bone samples were fixed in 4% buffered formalin, decalcified with sodium formiate 0.76 M and formic acid 1.6 M (Panreac Quimica, Barcelona, Spain) for 21 days, dehydrated in an ascending concentration series of ethanol, and embedded in paraffin. Sections 5 µm thick were cut with a microtome, mounted on slides, and stained with haematoxylin and eosin (Figure 12a). Morphometric measurements were performed on a digital photomicrograph of the sample at 10× magnification using dedicated analytic software (ImageJ 1.33, National Institute of Health, Bethesda, USA). Measurements, performed on the whole slide, were used to calculate the ratio of either the newly formed bone or the residual biomaterial areas within the whole tissue sample. Ratios are expressed as percentages. 

The histological analyses showed the presence of large acidophilic regions corresponding to vital bone. Indeed, higher magnification revealed the presence of both osteoblasts, along the edge of the tissue (Figure 12b, black arrows), and osteocytes inside the lacunae of ossified bone (Figure 12b, red arrows). Some biomaterial particles could be observed that appeared to still be remodeling (Figure 12a, black arrows). No inflammatory cell infiltrate was found. Histomorphometric measurements were as follows: newly formed bone, 39%; residual graft, 10%; medullar spaces, 51%. Histological samples were also observed under polarized light, highlighting regions with a more mature tissue organization as white birefringent areas (Figure 12c).

**Figure 13:**
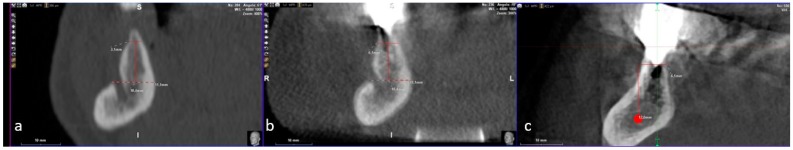
CT scans (element 45) performed before (**a**), 2 years after (**b**), and 6.5 years after implant rehabilitation. Before guided bone regeneration (**a**), the crestal bone thickness was only 3.1 mm. After the rehabilitation, during the follow-up period, the crestal bone thickness was maintained: it only decreased from 6.4 mm (**b**) to 6.1 mm (**c**).

Three and a half months after implant positioning, a three-crown provisional prosthesis was delivered, and, after one additional month, the definitive restoration enabled final rehabilitation. The patient was recalled every six months for clinical and radiographic follow-up, the latter being carried out by collecting endoral radiographs. After 2 and 6.5 years, the patient underwent other bone augmentation surgeries in other quadrants, which required recording additional CT scans. To investigate the post-augmentation ridge thickness variation over time, the pre-surgical and two additional scans were therefore used to measure ridge thickness; this was assessed at a fixed distance from the inferior alveolar nerve emergence. Six years after implant positioning, the CT scans showed that sufficient bone thickness at the recipient site was retained. Indeed, it was found to be of about 6.1 mm compared to 3.1 mm measured before the augmentation surgery (Figure 13).

**Figure 14:**
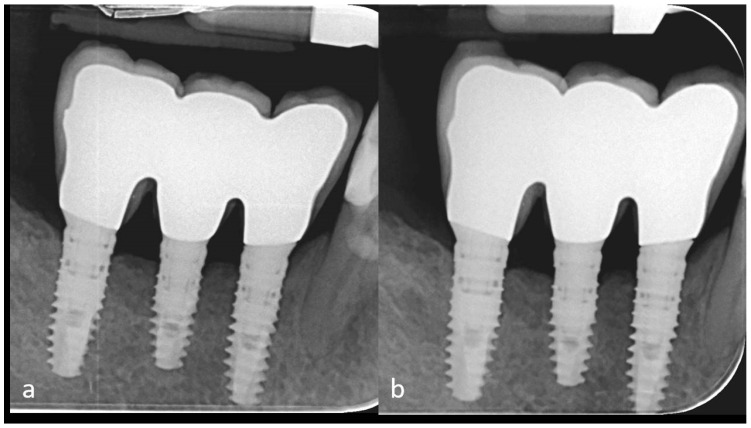
Intraoral radiographies performed 4 years (**a**) and 6.5 years (**b**) after implant rehabilitation show good maintenance of the peri-implant bone level.

The endoral radiography recorded at the follow-up visit at six and a half years showed minimal bone resorption around the implants (Figure 14). Throughout the follow-up period, the oral status indices FMPS and FMBS remained unaltered, the patient being highly motivated and undergoing periodical (four-monthly) oral hygiene treatments.

The patient was treated according to standard operative protocols and procedures that were not changed for the purpose of data collection and that did not result in any additional biological damage or discomfort to the patient: given this condition, according to the Italian National laws and to Good Clinical Practices concerning investigations with medical devices (i.e., ISO14155), the study did not require the approval of an ethics committee. The patient was made aware before surgery that a bone biopsy may be collected during implant placement for the purpose of clinical investigation and that such an operation cannot create any additional risk with respect to standard practice; thus, he signed an appropriate and explicit informed consent form.

## 3. Discussion

Although they show a right–left symmetric distribution within the maxilla and mandible, bone volume defects are more common in the posterior than in the anterior jaw regions [22]. Both their prevalence and severity correlate with age and, when particularly extensive, they can seriously compromise the possibility of positioning dental implants [23,24]. This is particularly true when the treatment is prosthetically driven, i.e., the implant placement is designed to fulfill esthetic and prosthetic requirements [25]. In such circumstances, restoring the alveolar bone volume with augmentation techniques is essential.

Among the various procedures available, GBR has become a very common approach, having the dual advantage that it can be used both in vertical and horizontal ridge augmentations and, if necessary, simultaneously with implant placement. Proper flap closure with no residual tension at the suture sites is a critical step for achieving clinical success, together with the use of long-lasting protective barriers that must not collapse into the underlying regenerating site [26,27].

Titanium meshes are particularly suitable for this application thanks to their being nonresorbable, rigid, and highly resistant, thus preventing the soft tissue from invading the regenerating site and collapsing in on it. At the same time, they are easy to handle and can be bent, cut, and adapted in size and shape according to the tridimensional architecture of the augmented alveolar ridge. Once shaped, the mesh can maintain its form over time, avoiding compression or displacement of the underlying graft material for the entire duration of the healing process. Titanium meshes have been shown to provide good clinical results when applied together with autogenous bone positioned under the membrane as an active filler promoting the regeneration of the augmented site [14,21,28]. Other authors have also demonstrated their successful use with deproteinized bovine bone alone or mixed with a fraction of autogenous bone [15,29,30].

The use of a titanium mesh for guided bone regeneration normally involves shaping the mesh during the surgery. After elevating the flap and positioning the graft material on the defect, the surgeon gives the mesh the appropriate shape, often by a trial-and-error process the ends only when the mesh has the appropriate shape. This leads to increased surgical time that may result in more discomfort for the patient and a higher risk of complications such as infection or swelling. Moreover, it introduces an additional element of variability into the surgery since the operation is carried out directly on the patient.

The present case report describes the rehabilitation of a right atrophic posterior mandible with a modified GBR procedure in which the mesh modeling was an integral part of the rehabilitation and surgical planning. In addition to defining the implant features, positioning, and inclination, a stereolithographic model of the patient’s mandible was prepared to enable shaping of the titanium mesh in advance. For a better fit with the clinical setting, a light-curing resin was used to simulate the filling of the osseous defect with the graft material and the restoration of the crestal bone volume. The titanium mesh was then preshaped on the model and sterilized by autoclaving to make it ready for use in surgery. In the authors’ experience, this simple operation makes the GBR procedure both faster, as the mesh modeling is performed before the surgery, and more predictable, since it allows for strict planning and control of a step that is generally performed without any preparatory phase. This is particularly critical during the rehabilitation of an atrophic posterior jaw, given the limited accessibility of the posterior sectors of the mouth.

The initial increase in ridge thickness that could be achieved in the present case report is smaller than that usually reported in published prospective and retrospective investigations involving augmentation through GBR and titanium meshes. Pieri et al. [15] or Sagheb et al. [31], for example, achieved greater (4.16 ± 0.59 mm and 5.5 ± 1.9 mm, respectively) initial horizontal augmentation, yet their series included also patients whose defects were less extended, that is, included fewer (1 or 2) elements than that of the present case report. The results of the present case report concerning implant success are consistent with those observed by Poli et al. [32] over a similar long-term follow-up period.

An equine-derived xenograft was used as grafting material under the titanium mesh. This graft is enzymatically treated to make it free of xenogeneic antigens while preserving bone collagen in its native conformation, along with its mechanical and biological properties [33,34,35,36,37,38,39,40]. This seems to confer to the graft remodeling kinetics comparable to that of natural bone; indeed, very few residual biomaterial particles could be identified by histological examination, implying that this equine-derived xenograft can be expected to be nearly completed resorbed 6.5 years after grafting. This result is consistent with the present clinical evidence on this bone substitute [41,42,43,44,45,46,47,48,49,50].

The remodeling kinetics of the equine-derived xenograft used in the present study, possibly leading to new bone formation in a physiologic time span, seems to be different from that of other xenografts undergoing other antigen-eliminating processes. Indeed, in a comparative clinical study performed on patients subjected to sinus lift elevation, enzyme-treated equine bone displayed a higher remodeling rate when compared to anorganic bovine bone (ABB), resulting in a greater quantity of newly formed bone [51].

ABB differs from the material used in this study in terms of the animal source and the manufacturing step of antigen removal, which is based on high temperatures. This treatment leads to the complete loss of the bone collagen matrix [52]. Thanks to its good osteoconductive properties, ABB is one of the most commonly used grafting materials and the one with the longest history of clinical application. However, its resorption properties may be less than ideal for implant placement as many independent studies have reported that residual ABB material persists in the recipient site even years after the grafting surgery [53,54,55,56,57]. Consistently, in an in vitro setting it was shown that human osteoclasts have reduced adhesion and bone-resorbing activity when cultured on ABB compared to bovine bone [58], suggesting that the slow remodeling kinetics is due to impaired interaction with the cells that play a major role in bone resorption and homeostasis. In a later study performed under the same experimental conditions, osteoclastic activity was also tested on enzymatically treated equine bone, and cells were found to adhere more efficiently and be more active compared to what was observed on ABB [59]. This different cellular behavior could presumably be attributed to the collagen-preserved features of the equine bone, given that osteoclasts recognize and bind native collagen [60].

A limitation of this study is that staining procedures other than haematoxylin and eosin (e.g., toluidine blue) might have allowed better visualization and a more detailed investigation of mineralized bone formation. Yet, the histologic results of this study seem to confirm the good remodeling properties of this graft, which may have been enhanced by the addition of the autogenous bone. Indeed, five months after the graft procedure, histomorphometric evaluation at the level of the regenerated site revealed that 39% was replaced by newly formed bone including regions with collagen fibers organized in lamellae. The augmented bone allowed the successful positioning and loading of three implants and the functional rehabilitation of the patient in a safe and lasting way.

## 4. Conclusions

In the present case report, a preshaped titanium mesh, in association with an enzyme-treated equine bone substitute, provided effective bone regeneration in a GBR augmentation procedure of an atrophic posterior mandible. The equine substitute allowed for effective new bone formation, confirming that this graft displays good remodeling properties.
